# Supplementary material for: Vitamin B6 deficiency disrupts serotonin signaling in pancreatic islets and induces gestational diabetes in mice
Source: Commun Biol. 2021 Mar 26;4:421. doi: 10.1038/s42003-021-01900-0 (PMC7998034; doi:10.1038/s42003-021-01900-0)
Supplement: Supplementary file 2 — Supplementary Information [file 42003_2021_1900_MOESM2_ESM.pdf]

Vitamin B6 deficiency disrupts serotonin signaling in pancreatic islets and induces gestational diabetes in mice

Ashley M. Fields[1], Kevin Welle[2], Elaine S. Ho[3], Clementina Mesaros[3], and Martha Susiarjo[1*].

[1] *Department of Environmental Medicine, University of Rochester School of Medicine and Dentistry, Rochester, NY, USA;*

[2] *Mass Spectrometry Resource Laboratory, University of Rochester, Rochester, NY, USA;*

[3] *Department of Systems Pharmacology and Translational Therapeutics, University of Pennsylvania Perelman School of Medicine, Philadelphia, PA, USA;*

[*] *Correspondence: martha_susiarjo@urmc.rochester.edu*

Supplemental Table 1. Maternal weight throughout gestation

	Gestational Day (GD)								
	GD 0.5	GD 2.5	GD 4.5	GD 6.5	GD 8.5	GD 10.5	GD 12.5	GD 14.5	GD 16.5
Control	18.6 g ± 0.67	18.1 g ± 0.66	18.4 g ± 0.62	18.9 g ± 0.91	19.8 g ± 0.86	21.1 g ± 1.29	23.1 g ± 1.39	27.3 g ± 1.57	30.3 g ± 2.19
Vitamin B6 deficiency	18.3 g ± 1.36	17.9 g ± 1.31	18.1 g ± 1.46	18.7 g ± 1.35	19.4 g ± 1.42	20.4 g ± 1.41	21.8 g ± 1.43	24.0 g ± 0.90	26.9 g ± 1.41

Supplemental Table 1: Body weight data during pregnancy. Control and vitamin B6 deficient dams were weighed every two-day starting at GD 0.5 and until GD 16.5. Data are based on 5 mice per group and were analyzed by two-way, repeated measures, ANOVA. Values represent mean ± SD.

Supplemental Table 2. Two-day average food consumption during pregnancy

	Before Pregnancy	Gestational Day (GD)								
		GD 0.5 – 2.5	GD 2.5 – 4.5	GD 4.5 – 6.5	GD 6.5 – 8.5	GD 8.5 – 10.5	GD 10.5 – 12.5	GD 12.5 – 14.5	GD 14.5 – 16.5	GD 16.5 – 18.5
Control	4.89 g ± 0.402	4.44 g ± 0.777	5.09 g ± 0.597	5.17 g ± 0.528	5.44 g ± 0.506	5.76 g ± 0.452	5.62 g ± 0.698	7.11 g ± 0.732	7.68 g ± 0.140	8.25 g ± 1.37
Vitamin B6 deficiency	5.35 g ± 0.392	4.79 g ± 0.744	4.60 g ± 0.538	4.93 g ± 0.573	5.05 g ± 0.568	5.64 g ± 0.843	5.22 g ± 1.01	6.37 g ± 0.686	7.17 g ± 0.449	7.80 g ± 0.779

Supplemental Table 2: Food consumption during pregnancy. Food intake was measured before and during pregnancy for control and vitamin B6 deficient females. The before pregnancy data represent an average of two-day food consumption for the three-week exposure prior to time-mating. During pregnancy, data represent a two-day average food consumption starting at GD 0.5 and until GD 18.5. Data are based on 5 mice per group and were analyzed by two-way, repeated measures, ANOVA. Values represent mean ± SD.

Supplemental Table 3. Mouse chow formulation from ENVIGO

Formula (g/kg)	Control TD. 150561	Vitamin B6 Deficiency TD. 160738
Casein, vitamin free	200.0	200.0
L-Cystine	3.0	3.0
Corn Starch	397.386	397.386
Maltodextrin	132.0	132.0
Sucrose	100.0	109.7535
Corn Oil	70.0	70.0
Cellulose	50.0	50.0
Mineral Mix, AIN-93G-MX (940946)	35.0	35.0
Niacin	0.03	0.03
Calcium Pantothenate	0.016	0.016
Pyridoxine HCl	0.0070	0.0005
Thiamin (81%)	0.006	0.006
Riboflavin	0.006	0.006
Folic Acid	0.002	0.002
Biotin	0.0002	0.0002
Vitamin B12	0.025	0.025
Vitamin E	0.15	0.15
Vitamin A	0.008	0.008
Vitamin D3	0.002	0.002
Vitamin K1	0.0008	0.0008
Choline Bitartrate	2.5	2.5
Tertiary butylhydroquinone, antioxidant	0.014	0.014

Selected Nutrient Information	Control	Vitamin B6 Deficiency
Protein (% kcal)	19.4	19.4
Carbohydrate (% kcal)	63.8	63.8
Fat	16.7	16.7
kcal/g	3.8	3.8

Supplemental Table 3: Formulation for the control (TD. 150561) and vitamin B6 deficiency (TD. 160738) diets. The level of pyridoxine HCL, an isoform of vitamin B6, differed between control and vitamin B6 deficiency diets. Information was adapted from ENVIGO datasheet.

Supplemental Figure 1

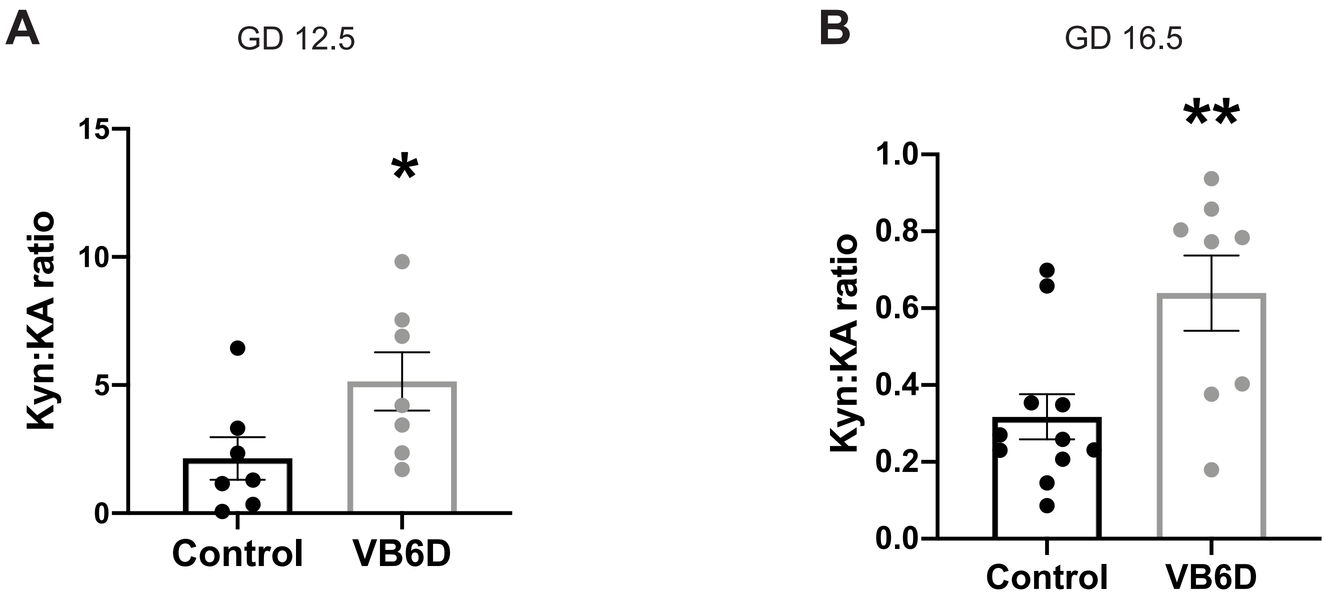

Supplemental Figure 1: LC-HRMS metabolite analysis for indirect measurement of vitamin B6 status during pregnancy. To indirectly test vitamin B6 status, the ratio of kynurenine (Kyn) and kynurenic acid (KA) in control and vitamin B6 deficient mice maternal liver is measured at GD 12.5 (A) and 16.5 (B). Data represent 6 – 8 mice per treatment group. Analysis of panels A and B done by an unpaired, two-sided, t-test. * P value ≤ 0.05, ** P value ≤ 0.01.

Supplemental Figure 2

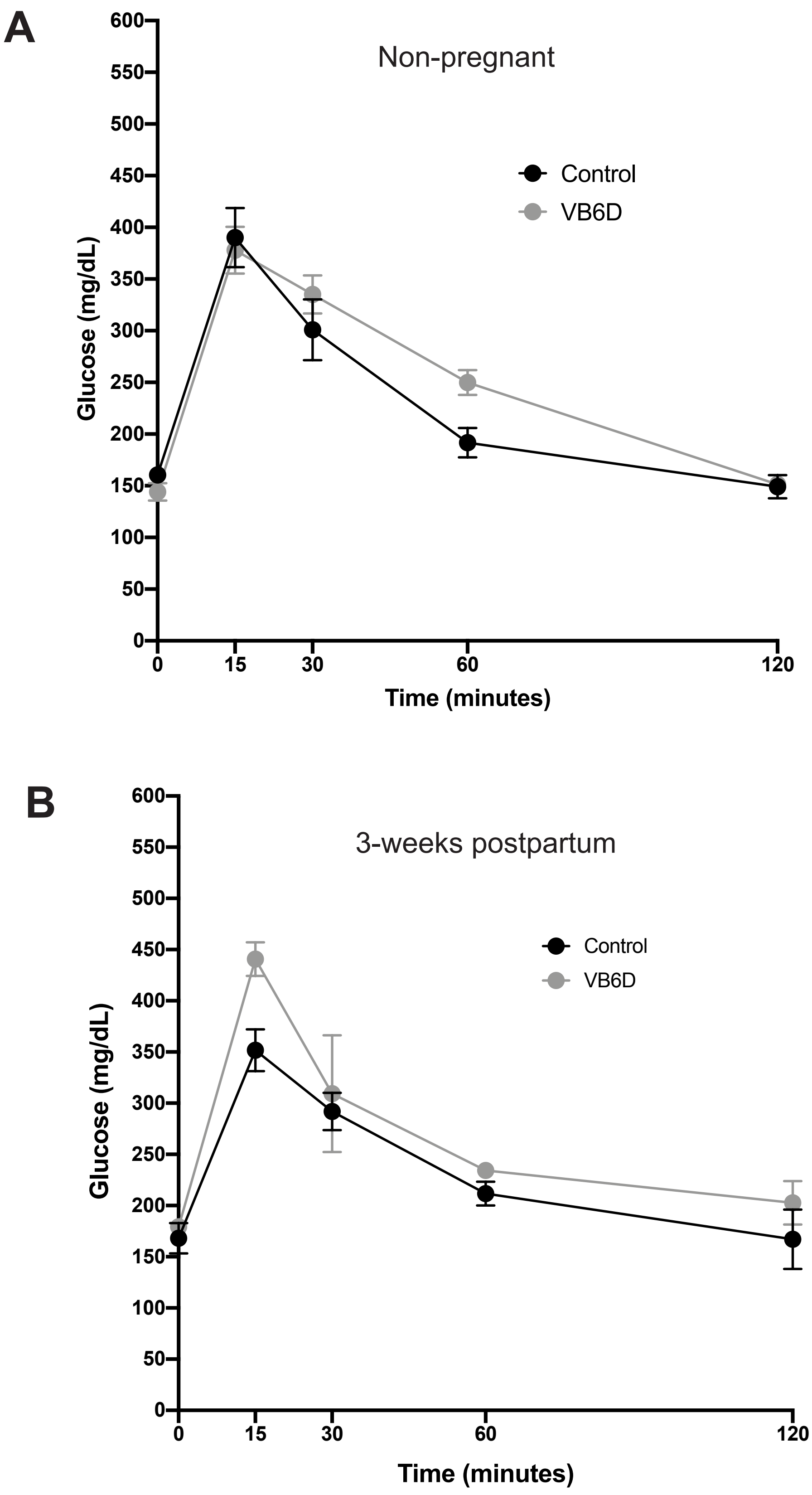

<u>Supplemental Figure 2</u>**: Vitamin B6 deficient non-pregnant and 3-week postpartum mice have normal glucose clearance.** Glucose time graphs are shown for non-pregnant mice (A) and 3-week post-partum dams (B). Data represent 7 (A) and 4 – 5 (B) dams per treatment group. All data were analyzed by a two-way repeated measures ANOVA.

Supplemental Figure 3

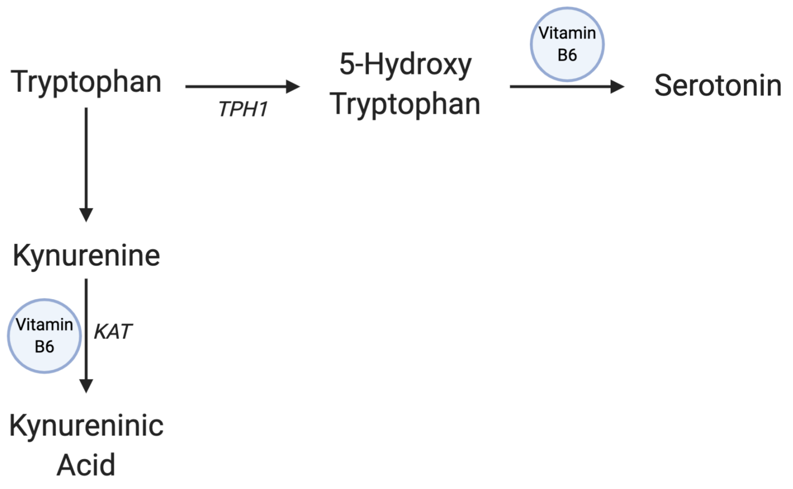

Supplemental Figure 3: Tryptophan catabolism requires vitamin B6 as a cofactor. Tryptophan has multiple catabolic fates; majority of tryptophan (~95 %) is converted into kynurenine, and subsequently kynurenic acid using kynurenine aminotransferase (KAT) as the enzyme. The other ~5 % of tryptophan is converted into 5-hydroxytryptophan by tryptophan hydroxylase 1 (TPH1), and subsequently catabolized into serotonin [2]. Both pathways are vitamin B6 dependent, as such production of downstream catabolites can be negatively impacted by vitamin B6 deficiency[2].

Supplemental Figure 4

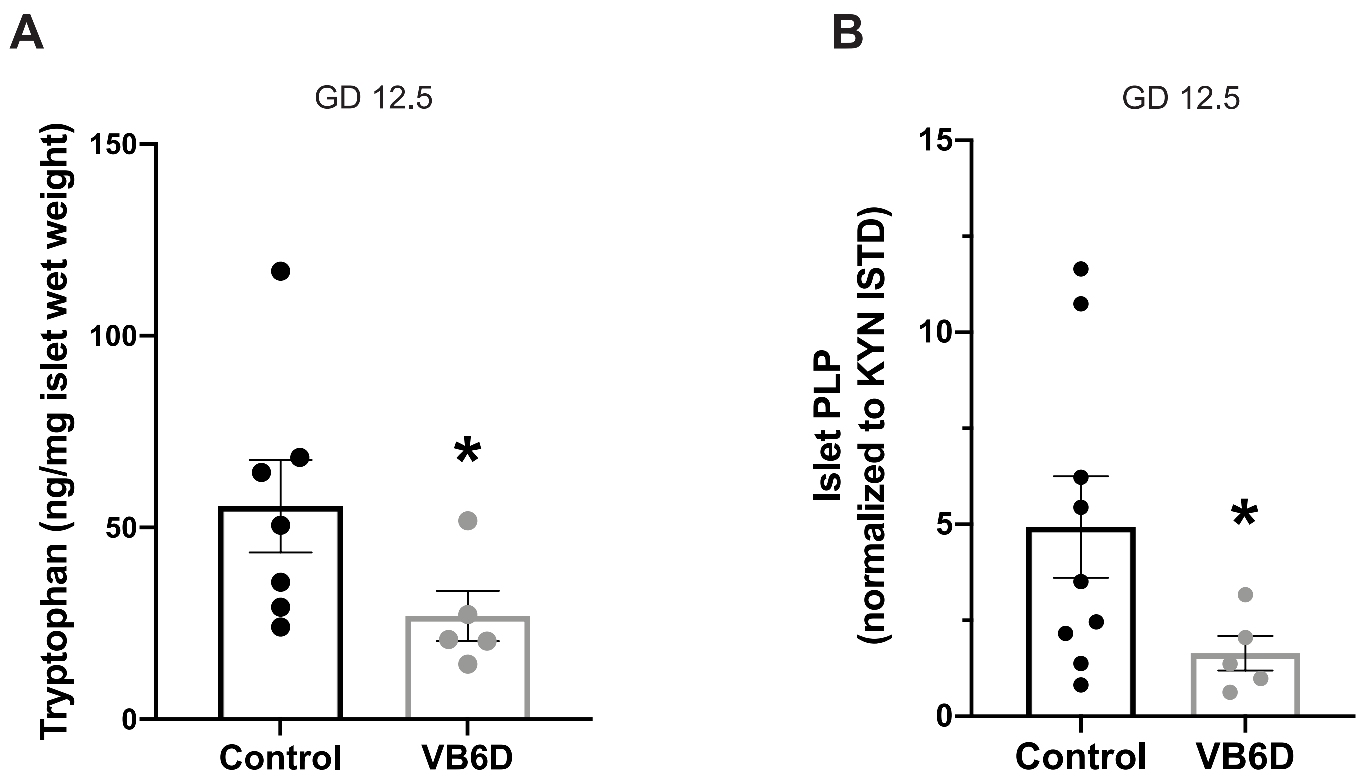

<u>Supplemental Figure 4</u>: Vitamin B6 deficient dams have reduced islet tryptophan and pyridoxine 5' phosphate (PLP) levels. Islets isolated from GD 12.5 vitamin B6 deficient dams have lower tryptophan (A) and reduced PLP levels (B) relative to controls. Data represents 6 – 10 mice per group. Unpaired, two- and one-sided t-tests were performed in panels A and B, respectively. * P values ≤ 0.05.

Supplemental Figure 5

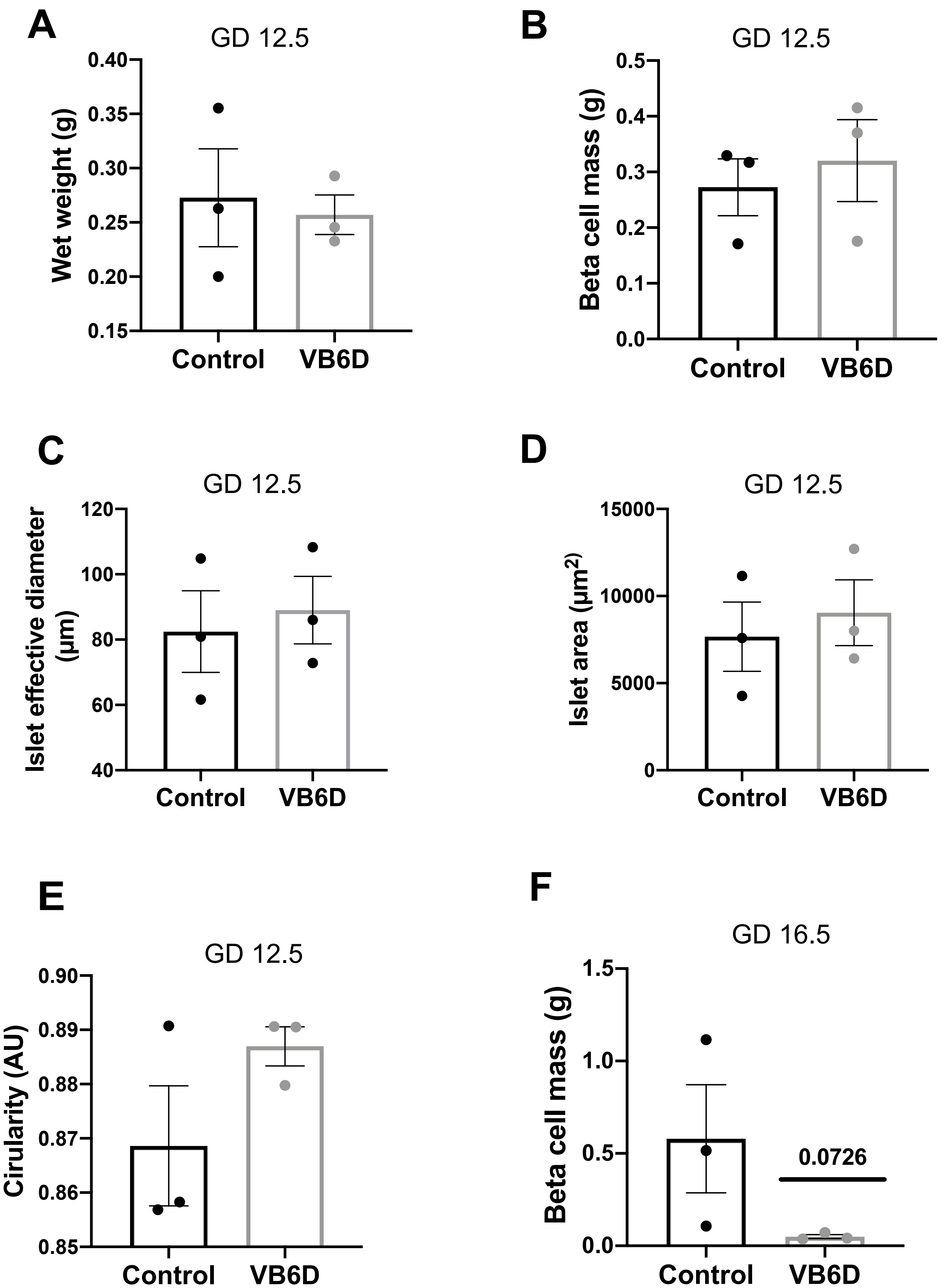

Supplemental Figure 5: Vitamin B6 deficiency does not impact β-cell morphology or mass during pregnancy. Shown in panels A to E are different islet characteristics. Unpaired, two-sided, t-tests reveal, at GD 12.5, there are no significant differences observed in pancreas wet weight (A), beta cell mass (B), islet effective diameter (C), islet area (D), nor islet circularity (E) in pancreases from control and vitamin B6 deficient (VB6D) dams. There was, however, a trending decrease in β-cell mass at GD 16.5 (F). Data are based on 40 – 50 islets per mouse with 3 mice total.

Supplemental Figure 6

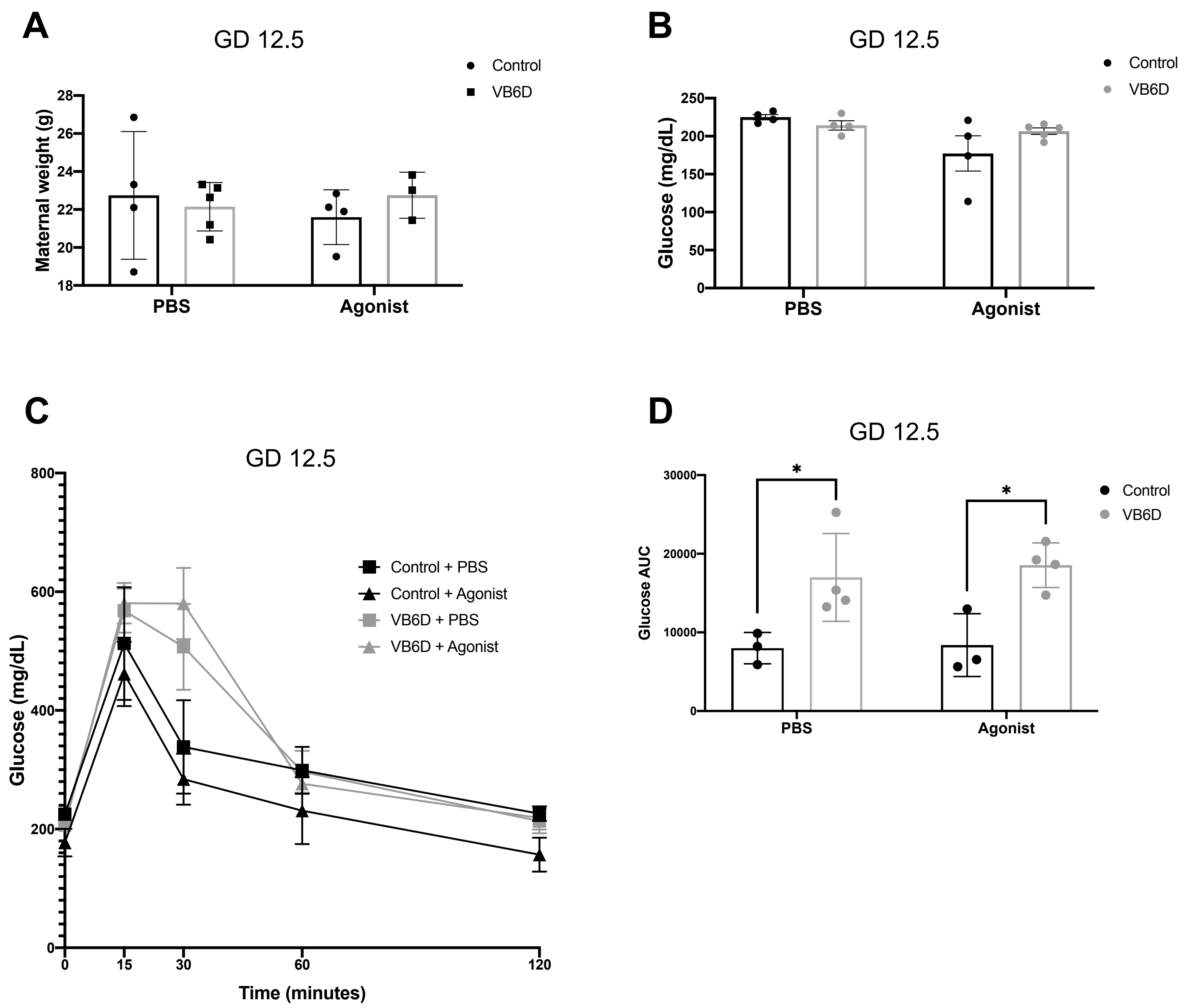

<u>Supplemental Figure 6</u>: **Vitamin B6 deficient mice are glucose intolerant at GD 12.5 and treatment with HTR2B agonist do not change glucose tolerance.** Diet and/or HTR2B agonist treatment did not influence maternal weight (A) or fasting glucose (B). Shown in (C) is the glucose time graphs for all four treatment groups. Glucose area under curve (AUC) analysis for PBS and HTR2B agonist treated GD12.5 control and vitamin B6 deficient (VB6D) dams (D) revealed a statistically significant diet effect, using a two-way ANOVA. Furthermore, no significant interaction between diet and agonist treatment was detected. Additional unpaired, two-sided, t-test analysis comparing differences in effects of vitamin B6 deficiency in both the PBS- and agonist-treated groups show that VB6D dams are glucose intolerant regardless of treatment. Panels A and B were analyzed by two-way ANOVA and panel C by two-way, repeated measures ANOVA. Data represent 4 – 5 mice * $P \leq 0.05$

Supplemental Figure 7

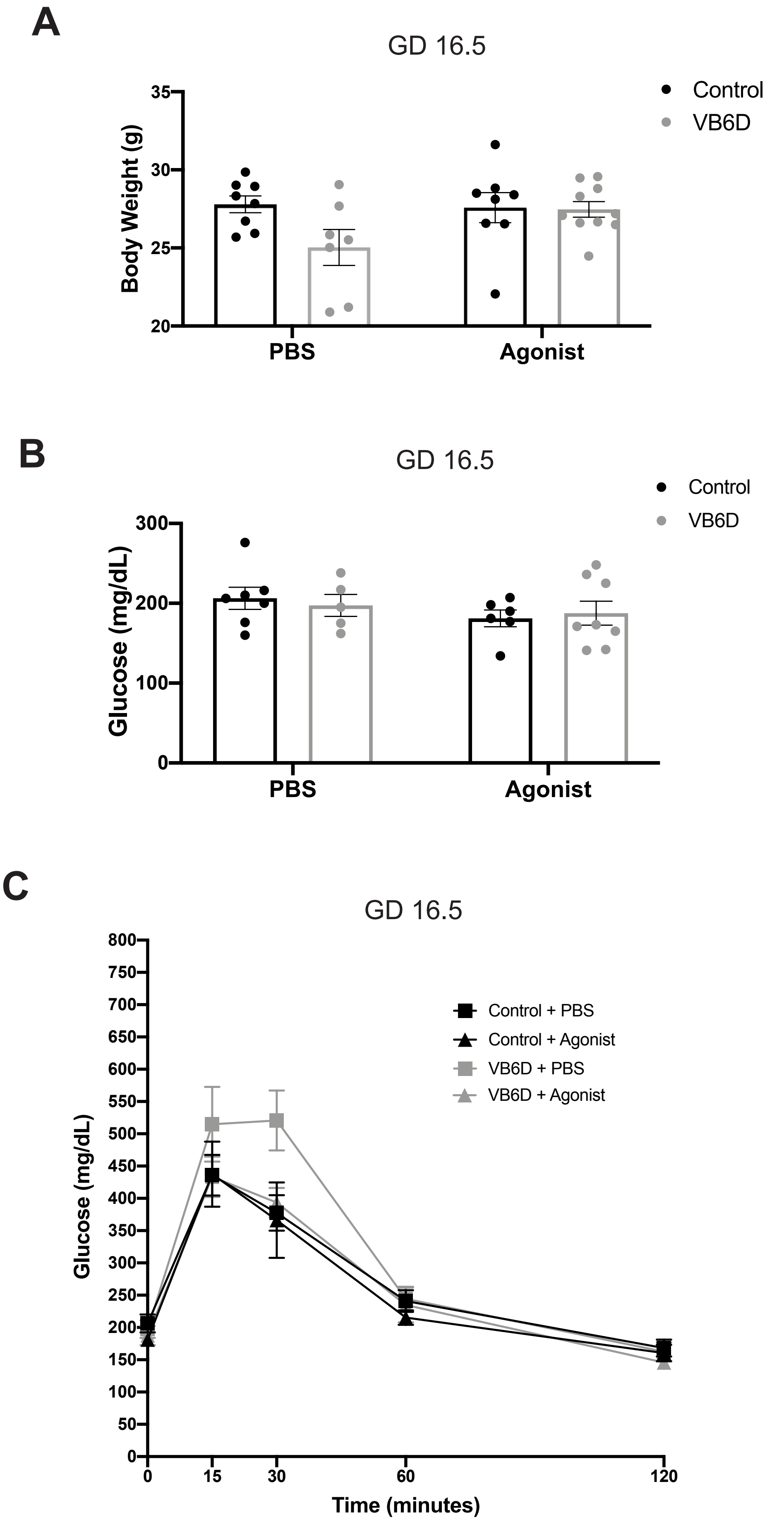

Supplemental Figure 7

Supplemental Figure 7: HTR2B agonist treatment improves glucose tolerance at GD 16.5. Diet and/or HTR2B agonist treatment with did not influence maternal weight (A) or fasting glucose (B). Shown in (C) is the glucose time graphs for all four treatment groups of PBS and HTR2B agonist treated GD 16.5 control and vitamin B6 deficient (VB6D) dams. Panels A and B were analyzed by two-way ANOVA and panel C by two-way, repeated measures ANOVA. Data represent 5 – 7 mice.

Supplemental Figure 8

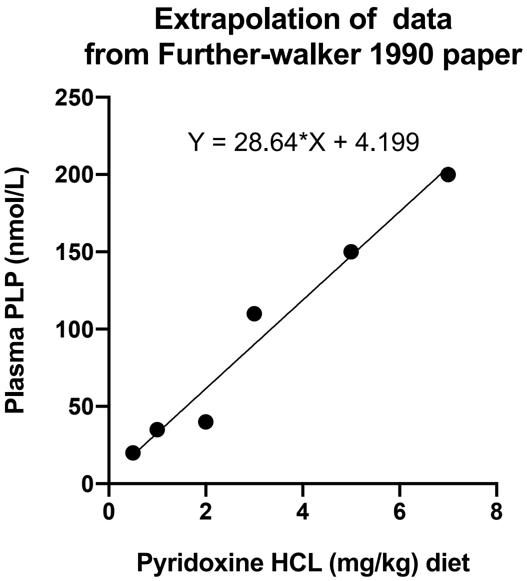

Pyridoxine HCL (mg/kg)	PLP (nmol/L)
0.50	20.0
1.00	35.0
2.00	40.0
3.00	110.0
5.00	150.0
7.00	200.0

Supplemental Figure 8: Creation of vitamin B6 deficient diet based on data extrapolation from Further-Walker et al.,[1]. Shown are the estimated values of pyridoxine HCl (PL HCl) in diet (X axis and left column) and the correlating pyridoxal 5' phosphate (PLP) in plasma (Y axis and right column), recreating Figure 1 from the Further-Walker et al.,[1].. Linear regression analysis provides a line (Y = 28.64*X + 4.199) that best fits the data. Additionally, we expect our vitamin B6 deficiency diet (0.5 mg/kg PL HCl) to achieve PLP blood plasma of 18.5 nmol/L, which is below the vitamin B6 deficiency threshold in humans (< 20 nmol/L).

Supplemental Figure 9

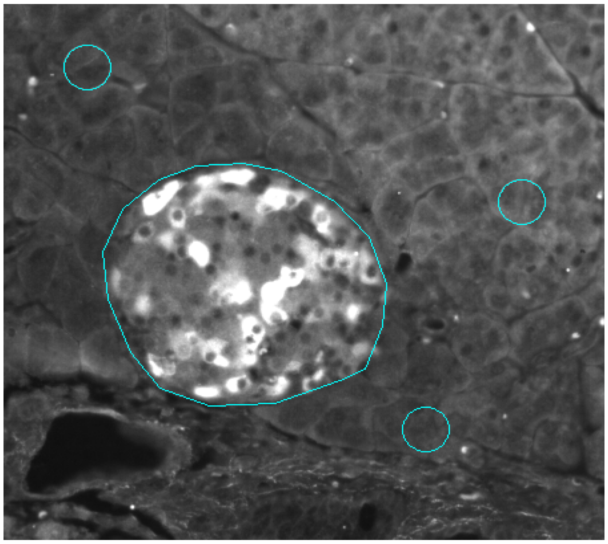

	Area	Mean	IntDen	RawIntDen
1	127167	9383.842	1193315062	1193315062
2	3852	4604.117	17735058	17735058
3	3852	6262.255	24122208	24122208
4	3852	4607.605	17748494	17748494

CTCF = Intergrated Density of traced islet –
(area of traced islet X
average mean grey value of background)

<u>Supplemental Figure 9</u>: Representative image of islet serotonin quantification. Shown is a representation of an islet analysis using ImageJ. Quantification features in ImageJ measure the islets area and integrated density (IntDen). Additionally, the average of 3 equal size regions adjacent to the islet reveals the mean grey value of background (Mean). Further analysis by corrected total cell fluorescence (CTCF), using the following equation: CTCF = integrated density of traced islet – (area of traced islet x average of mean gray value of background readings), provides the serotonin quantification for each islet[3].

Supplemental Figure 10

A

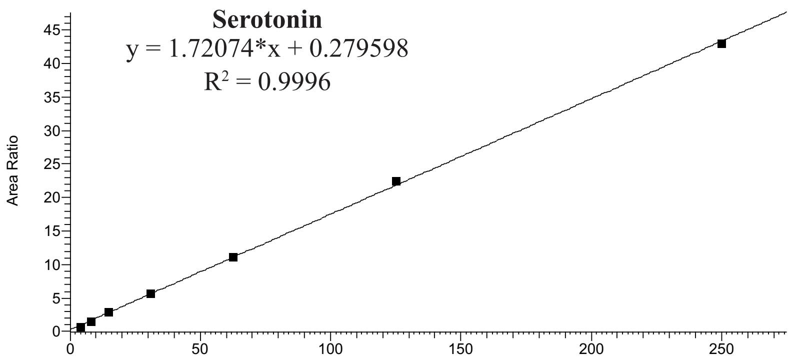

B

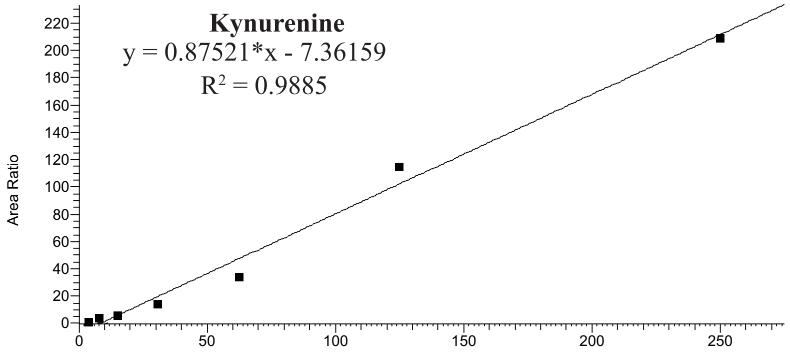

<u>Supplemental Figure 10</u>: LC-HRMS internal standard calibration curves for serotonin and kynurenine. Calibration curves were generated for serotonin (A) and kynurenine (B) internal standards by running a range of internal standard concentrations and measuring their peak area readout from the LC-HRMS. The x- and y- axes represent the retention time (minutes) and relative abundance of internal standard, respectively. Linear regression analysis was performed to produce an equation for absolute metabolite calculations.

Supplemental References

1. Furth-Walker, D., D. Leibman, and A. Smolen, *Relationship between blood, liver and brain pyridoxal phosphate and pyridoxamine phosphate concentrations in mice.* J Nutr, 1990. **120**(11): p. 1338-43.
2. Ueland, P.M., et al., *Direct and Functional Biomarkers of Vitamin B6 Status.* Annu Rev Nutr, 2015. **35**: p. 33-70.
3. McCloy, R.A., et al., *Partial inhibition of Cdk1 in G 2 phase overrides the SAC and decouples mitotic events*. Cell Cycle, 2014. **13**(9): p. 1400-12.
