# Supplementary material for: Vitamin B6 deficiency disrupts serotonin signaling in pancreatic islets and induces gestational diabetes in mice
Source: Commun Biol. 2021 Mar 26;4:421. doi: 10.1038/s42003-021-01900-0 (PMC7998034; doi:10.1038/s42003-021-01900-0)
Supplement: Supplementary file 3 — Description of Additional Supplementary Files [file 42003_2021_1900_MOESM3_ESM.pdf]

Description of Additional Supplementary Files

Files name: Supplementary Data 1

Description: Source data underlying plots shown in figures.
