# Supplementary material for: Vitamin B6 deficiency disrupts serotonin signaling in pancreatic islets and induces gestational diabetes in mice
Source: Commun Biol. 2021 Mar 26;4:421. doi: 10.1038/s42003-021-01900-0 (PMC7998034; doi:10.1038/s42003-021-01900-0)
Supplement: Supplementary file 5 — Reporting Summary [file 42003_2021_1900_MOESM5_ESM.pdf]

nature research

Corresponding author(s): Ashley M. Fields
Dr. Clementina Mesaros
Elaine S. Ho
Kevin Welle
Dr. Martha Susiarjo

Last updated by author(s): Jan 11, 2021

Reporting Summary

Nature Research wishes to improve the reproducibility of the work that we publish. This form provides structure for consistency and transparency in reporting. For further information on Nature Research policies, see our Editorial Policies and the Editorial Policy Checklist.

Statistics

For all statistical analyses, confirm that the following items are present in the figure legend, table legend, main text, or Methods section.

n/a	Confirmed	
☐	☒	The exact sample size (n) for each experimental group/condition, given as a discrete number and unit of measurement
☐	☒	A statement on whether measurements were taken from distinct samples or whether the same sample was measured repeatedly
☐	☒	The statistical test(s) used AND whether they are one- or two-sided<br>*Only common tests should be described solely by name; describe more complex techniques in the Methods section.*
☐	☒	A description of all covariates tested
☐	☒	A description of any assumptions or corrections, such as tests of normality and adjustment for multiple comparisons
☐	☒	A full description of the statistical parameters including central tendency (e.g. means) or other basic estimates (e.g. regression coefficient) AND variation (e.g. standard deviation) or associated estimates of uncertainty (e.g. confidence intervals)
☒	☐	For null hypothesis testing, the test statistic (e.g. F, t, r) with confidence intervals, effect sizes, degrees of freedom and P value noted<br>*Give P values as exact values whenever suitable.*
☒	☐	For Bayesian analysis, information on the choice of priors and Markov chain Monte Carlo settings
☒	☐	For hierarchical and complex designs, identification of the appropriate level for tests and full reporting of outcomes
☒	☐	Estimates of effect sizes (e.g. Cohen's d, Pearson's r), indicating how they were calculated

Our web collection on statistics for biologists contains articles on many of the points above.

Software and code

Policy information about availability of computer code

Data collection	For microscopy: Images were acquired using an Upright ECLIPSE Ni-E microscope (Nikon) equipped with the NIS-Elements-Advance Research imaging software. Additional features in NIS-Elements (including cell counting, area measurement, and conversion to Tiff files) were used. After the images were converted to grey-scale Tiff files, ImageJ (v1.48, NIH) was used to quantify fluorescent intensity for islet serotonin.<br><br>For liquid chromatography-mass spectrometry: Peak areas were calculated using LC Quan node of the XCalibur software (ThermoFisher).<br><br>For liquid chromatography high resolution mass spectrometry: Calibration curves were constructed for all of the analytes using authentic standards and same amounts of internal standards as used for the islet samples. The area ratio of analyte to internal standard was plotted versus the amount of analyte and a linear regression was used to calculate the amount of metabolite in the tube. The calculated amount was adjusted to the wet weight of the islets.
Data analysis	All statistical analyses were performed using GraphPad Prism 8 (v8.3.0)

For manuscripts utilizing custom algorithms or software that are central to the research but not yet described in published literature, software must be made available to editors and reviewers. We strongly encourage code deposition in a community repository (e.g. GitHub). See the Nature Research guidelines for submitting code & software for further information.

Data

Policy information about availability of data

All manuscripts must include a data availability statement. This statement should provide the following information, where applicable:

- Accession codes, unique identifiers, or web links for publicly available datasets
- A list of figures that have associated raw data
- A description of any restrictions on data availability

The authors declare that the data supporting the findings of this study are available within the paper and its supplementary information files. All data are available from the corresponding author upon request. Additionally, Supplemental data 1 contains the source data for each main figure and panels.

Field-specific reporting

Please select the one below that is the best fit for your research. If you are not sure, read the appropriate sections before making your selection.

☒ Life sciences ☐ Behavioural & social sciences ☐ Ecological, evolutionary & environmental sciences

For a reference copy of the document with all sections, see nature.com/documents/nr-reporting-summary-flat.pdf

Life sciences study design

All studies must disclose on these points even when the disclosure is negative.

Sample size

We did a calculation to predetermine sample size for the glucose tolerance testing based on our preliminary data. Based on this calculation, we would need 17 mice per treatment group to achieve 80% power (alpha = 0.05; two-sided). In the actual experiments described in Figures 1 and 2, however, the variance within the treatment groups was significantly smaller than the preliminary data and we achieved statistical significance using only 6 to 8 mice.

Sample size for immunofluorescence (Figures 3B and 4B) was performed using 3 mice per treatment group and 40 to 50 islets were analyzed per mouse (approximately 6000 beta cells). These sample sizes are consistent with published histological rodent pancreatic studies that typically analyze a minimum of 30 islets per mouse or 3,000 beta cells.

For serum serotonin quantification (Figure 3C), we analyzed serum samples from 5 to 7 gestational day 16.5 pregnant females per treatment group for liquid chromatography-mass spectrometry. We did not perform any sample size calculations or power analysis prior to the analysis. We selected to use sample size similar to our glucose tolerance studies and the tested sample size was sufficient to achieve statistical significance.

For indirect measure of vitamin B6 status in Figure 1A, we analyzed 6 to 8 maternal liver samples from gestational day 12.5 pregnant females per treatment group for high resolution-mass spectrometry. We did not perform any sample size calculations or power analysis prior to our results, but the data was sufficient to achieve statistical significance.

Data exclusions

In Figure 1, there were some data excluded from the glucose tolerance studies. Upon completion of glucose tolerance testing, the gestational day 12.5 and 16.5 pregnant mice were euthanized and examined for internal damage from the intraperitoneal glucose injection. If the mouse had internal bleeding or a ruptured uterine sack from misguided injection, their blood glucose values were excluded, because this could cause confounding increase in glucose due to stress-related damage or decreased glucose due to injecting into the uterine sack. These exclusion criteria were pre-established prior to the conducting the experiment.

Replication

Our glucose tolerance tests were conducted using multiple cohorts and reproducible findings were consistently observed. Additionally, we used female mice of similar age across treatment groups and the glucose tolerance testing was performed at the same time every time, in the same location, and performed by the same individual. For the immunohistochemistry, all slides were stained using master mixes of primary and secondary antibodies. Images were captured using identical exposure and laser intensity settings.

Randomization

Six-to-eight-week old virgin female mice were randomly selected to treatment groups, ensuring that there was an similar distribution of ages across treatment groups. For studies involving the HTR2B agonist, we allocated randomly equal or similar number of females to either the PBS- or HTR2B-injected treatment group.

Blinding

All mice were given unique identification numbers. Data collection and data analysis were conducted throughout the study using these unique identification numbers. Complete blinding was not possible as the same person allocated the mice and performed data collection and data analysis. The exceptions were the experiments conducted using mass spectrometry; they were done in a blinded manner as the serum and liver samples sent to Drs. Mesaros and Welle were coded by their identification numbers and were only decoded when the data were analyzed

Reporting for specific materials, systems and methods

We require information from authors about some types of materials, experimental systems and methods used in many studies. Here, indicate whether each material, system or method listed is relevant to your study. If you are not sure if a list item applies to your research, read the appropriate section before selecting a response.

Materials & experimental systems

n/a	Involved in the study	
☐	☒	Antibodies
☒	☐	Eukaryotic cell lines
☒	☐	Palaeontology and archaeology
☐	☒	Animals and other organisms
☒	☐	Human research participants
☒	☐	Clinical data
☒	☐	Dual use research of concern

Methods

n/a	Involved in the study	
☒	☐	ChIP-seq
☒	☐	Flow cytometry
☒	☐	MRI-based neuroimaging

Antibodies

Antibodies used

Rabbit Recombinant anti-Insulin (1:200, Abcam; ab18547; Clone EPR17359; Lot # GR3210143-4)
Mouse anti-MCM-2 (1:750; ThermoFisher; MA5-15895; Clone 2B3; Lot # UJ2861441)
Rat anti-Serotonin (1:200; Abcam; ab6336; Clone YC5/45; Lot # GR3253852-10)
Donkey anti-Rabbit-AlexaFluor® 488 (1:200; JacksonImmuno; Cat # 711-545-152; Lot # 144217)
Donkey anti-Mouse-AlexaFluor® 647 (1:750; JacksonImmuno; Cat # 715-605-150; Lot # 139394)
Donkey anti-Rat-AlexaFluor® 647 (1:200; JacksonImmuno; Cat # 712-605-153; Lot # 142891)
DAPI (300nM; Invitrogen, Cat # D1306; Lot # 2031179)

Validation

Rabbit Recombinant anti-Insulin (1:200, Abcam; ab18547; Clone EPR17359; Lot # GR3210143-4): Validation studies can be found on Abcam images section. Abcam used pancreatic islets as a positive control and as a negative control they used PBS instead of the primary antibody and used the anti-rabbit Alexa Fluor® 594 at 1/1000 dilution and did not obtain signal detection via fluorescent microscopy. Additionally, our studies clearly show that the insulin antibody specifically localized to the pancreatic islets (where insulin-secreting beta cells are found).

Mouse anti-MCM-2 (1:750; ThermoFisher; MA5-15895; Clone 2B3; Lot # UJ2861441):
ThermoFisher has validated immunohistochemistry reactivity with mouse, rat, and human tissue, and used cancerous biopsy samples (i.e. highly proliferative) as their positive controls. Our lab has also tested the antibody on gestational day 9.5 whole gestational sack as a positive control as this tissue is highly proliferative. Additionally, we saw increased beta cell proliferation upon HTR2B treatment, the receptor responsible for beta cell proliferation, indicating that the antibody is a suitable proliferation marker.

Rat anti-Serotonin (1:200; Abcam; ab6336; Clone YC5/45; Lot # GR3253852-10):
Abcam validated that this antibody is highly effective in marking serotonergic neurons in the rodent brain (positive control). Our own study showed that it co-localized with the insulin antibody and was specific to the pancreatic islets.

Animals and other organisms

Policy information about studies involving animals; ARRIVE guidelines recommended for reporting animal research

Laboratory animals

Six-to-eight-week old virgin C57BL/6J female mice were primarily used in the study. We also used eight-to-ten-week old C57BL/6J male mice for time mating.

Wild animals

This study did not involve the use of wild animals.

Field-collected samples

This study did not involve samples collected from the field.

Ethics oversight

All mouse work was carried out with the approval of the University Committee on Animal Resources of the University of Rochester School of Medicine and Dentistry.

Note that full information on the approval of the study protocol must also be provided in the manuscript.
